# Supplementary material for: A genetically encoded tool for reconstituting synthetic modulatory neurotransmission and reconnect neural circuits in vivo
Source: Nat Commun. 2021 Aug 9;12:4795. doi: 10.1038/s41467-021-24690-9 (PMC8352926; doi:10.1038/s41467-021-24690-9)
Supplement: Supplementary file 1 — Supplementary Information [file 41467_2021_24690_MOESM1_ESM.pdf]

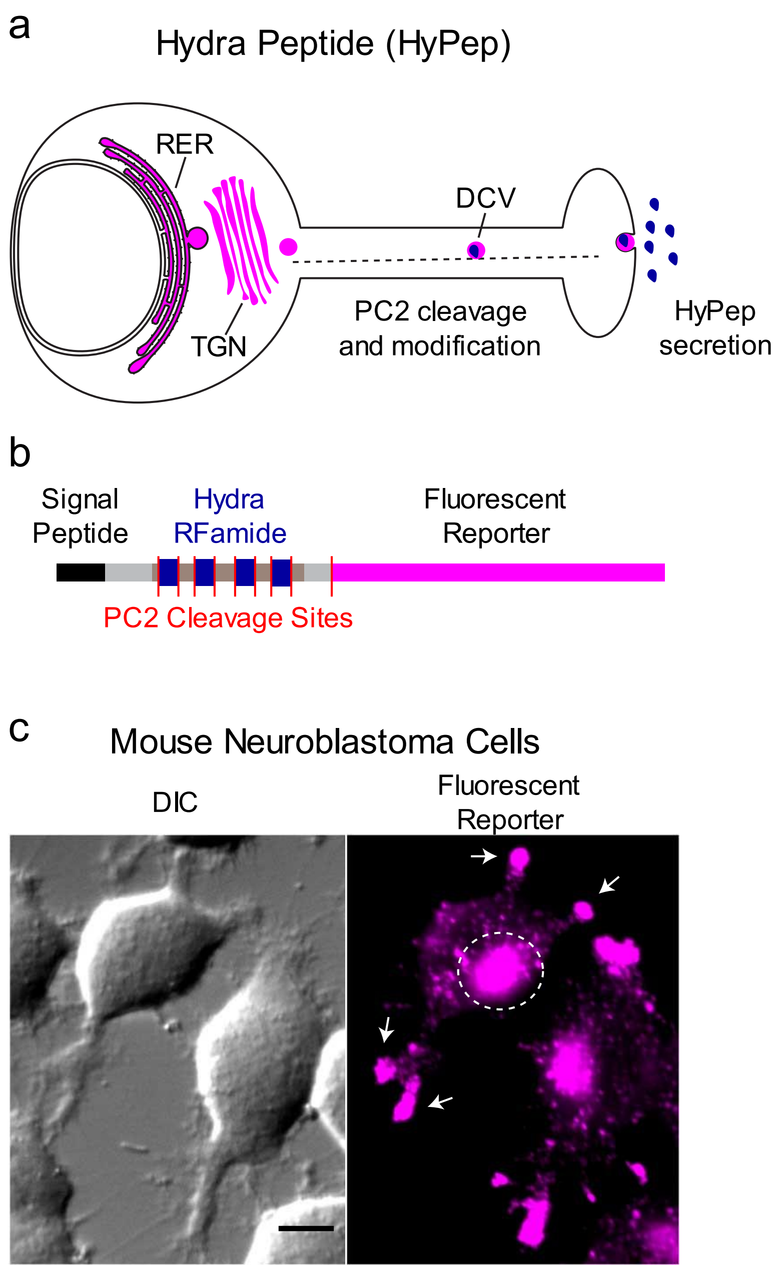

Figure S1

Supplementary Figure 1. *Hydra*-derived neuropeptide (HyPep) strategy for heterologous expression. a, Illustration of the cellular neuropeptide processing pathway utilized by the *Hydra*-derived neuropeptide (HyPep, blue). The regulated exocytosis pathway involves synthesis in the rough endoplasmic reticulum (RER, magenta), trafficking through the trans-golgi network (TGN), and packaging into dense-core vesicles (DCVs) for release. **b**, Schematic of the designed HyPep pre-pro-peptide carrier construct for heterologous expression of reconstitution of HySyn. An N-terminal signal peptide ('Signal Peptide'), based on the broadly expressed Neuropeptide Y sequence, targets the precursor molecule to the RER for synthesis. Cleavage at consensus recognition sites (red lines) for the endopeptidase pre-pro-convertase 2 (PC2) produces individual peptide fragments for further chemical modification into active neuropeptides ('*Hydra* RFamide'). **c**, Expression of the HyPep-GFP reporter (pseudocolored magenta) in Neuro2a neuroblastoma cells reveals localization in a pattern consistent with sites of neuropeptide synthesis and trafficking, including expression in compartments appearing to be perinuclear (as expected for the RER, white dashed circle) and at neurite extensions (white arrows; see also Fig 3 for *in vivo* localization of HySyn components). Scale bar indicates 10µm. Transfection and reporter expression (**c**) was observed reproducible, including in the 22 cases where successful electrophysiological recordings were made (see Fig S2) and 3 independent populations with GCaMP expression (See Fig 2). Source data are provided as a Source Data file.

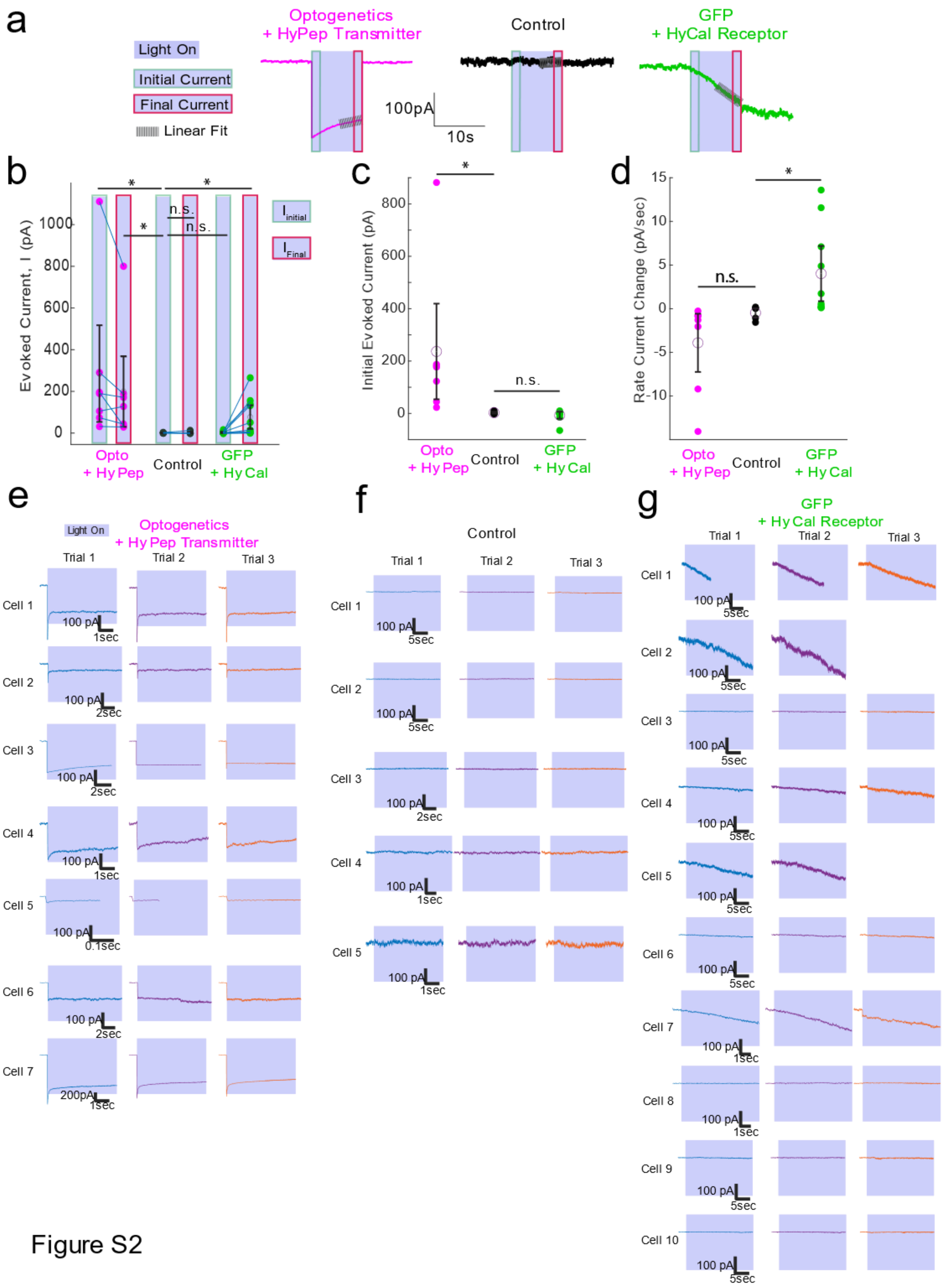
a
Light On
Initial Current
Final Current
Linear Fit
Optogenetics
+ HyPep Transmitter
Control
GFP
+ HyCal Receptor
100pA
10s
b
Evoked Current, I (pA)
I initial
I Final
n.s.
n.s.
Opto
+ Hy Pep
Control
GFP
+ Hy Cal
c
Initial Evoked Current (pA)
n.s.
d
Rate Current Change (pA/sec)
n.s.
e
Light On
Optogenetics
+ Hy Pep Transmitter
Trial 1
Trial 2
Trial 3
Cell 1
Cell 2
Cell 3
Cell 4
Cell 5
Cell 6
Cell 7
f
Control
g
GFP
+ Hy Cal Receptor
Cell 8
Cell 9
Cell 10

Figure S2

Supplementary Figure 2. 'Postsynaptic' currents in HyCal-expressing cells increase after stimulation of 'presynaptic' HyPep-expressing cells. a, Sample response profiles illustrating light-evoked currents in 'presynaptic' (magenta current trace on left, 'Optogenetics + HyPep Transmitter'); Control (black current trace in middle, 'Control'); and 'postsynaptic' (green current trace on right, 'GFP + HyCal Receptor') cells labeled as in Fig 1. Light stimulation (blue shading) evokes immediate currents by optogenetic as highlighted in the initial 100ms ('Initial Current', green box) that typically diminishes by the final 100ms ('Final Current', red box) of light stimulation, consistent with known desensitization properties[22]. No current was evoked in control cells that do not express the ChRoME optogenetic channel. In cells expressing the 'postsynaptic' HyCal receptor in the presence of HyPep-expressing cells that have been optogenetically stimulated, current increases over time, culminating in the final 100ms of the stimulus (red box, far right diagram). For subsequent quantifications, we used both an approach based on stimulus-locked time bins ('Initial Current' and 'Final Current' quantified in **b**) and linear fit of the current trace ('Linear Fit' quantified in **c** and **d**, represented in the schematic in **a** as region shaded grey in the graphs). **b,** Quantification of initial current ('$I_{Initial}$', green box) and final current ('I_{Final}', red box) within light stimulus-locked time bins as illustrated in **a.** Each dot in the quantification (**b-d**) represents the mean of the trials for a single cell (**e**). Optogenetically activated 'presynaptic' cells (magenta, 'Opto + HyPep') show evoked currents during both the initial window after stimulus ('$I_{Initial}$', in green box on left of magenta pair) and at the end of the stimulus ('I_{Final}', in red box on right of magenta pair) as compared to the control group ('Control', middle two samples in black, p=0.0125 for Control $I_{Initial}$ (n=5) vs Opto +HyPep $I_{Initial}$ (n=7), p=0.0125 for Control $I_{Initial}$ (n=5) vs Opto +HyPep I_{Final} (n=7)). Cells expressing the HyCal receptor ('GFP + HyCal', right two samples in green) do not show an initial current upon light activation ('$I_{Initial}$', on left of green pair in green box) but a current emerges by the end of stimulation ('I_{Final}', on right of pair in red box). p=1.0 for Control $I_{Initial}$ (n=5) vs Control I_{Final} (n=5), p=1.0 for Control $I_{Initial}$ (n=5) vs GFP + HyCal $I_{Initial}$ (n=10), p=0.04 for Control $I_{Initial}$ (n=5) vs GFP + HyCal I_{Final} (n=10). **c,** Fitting the current with a linear model (y=*m*x+*b*, gray dashed line in (**a**) indicates a significantly elevated initial current term (*b* term) with optogenetic stimulation (magenta) relative to control (black) or HyCal receptor (green) (p=0.0125 for Control (n=5) vs Opto + HyPep (n=7), p=1.0 for Control I (n=5) vs GFP + HyCal (n=10)). **d**, This initial elevation of current in optogenetically activated cells is reduced with continued stimulation as illustrated by a negative rate of change term (*m* term in y=*m*x+b) for optogenetically activated cells (magenta). In contrast, HyCal-expressing cells experience a current that rises with continued stimulation (green) (p=0.5305 for Control (n=5) vs Opto + HyPep (n=7), p=0.0235 for Control (n=5) vs GFP + HyCal (n=10)). These kinetics are consistent with known channel properties: Light stimulation provides near simultaneous activation of optogenetic tools followed by desensitization[22]. In contrast, HyCal responds to the concentration of HyPep-derived neuropeptide available, which would be expected to increase over time of optogenetic stimulation of HyPep-expressing cells, and which lacks desensitization[12]. **e,** Individual current traces (blue, purple, orange) for 'presynaptic' cells ('Optogenetics + HyPep Transmitter'). Each dot in the quantification (**b**-**d**) represents the mean of the trials for a single cell (shown in **e**-**g**). (**e**). **f,** Individual current traces (blue, purple, orange) for cells lacking reporter expression ('Untransfected Controls'). **g,** Individual current traces (blue, purple, orange) for cells expressing the 'postsynaptic' HyCal receptor ('GFP + HyCal Receptor') in the presence of co-cultured cells co-expressing HyPep and the optogenetic tool (Chrimson). The quantification represents the average value for technical replicates of each cell assayed. Note that light timing was manual, and some cells were stimulated for shorter periods of time, indicated in the scales and responses. Error bars indicate 95% confidence and * indicates p<0.05 from planned comparisons using two-tailed Mann-Whitney-Wilcoxon with Bonferroni multiple-testing correction against pre-stimulus control. Source data are provided as a Source Data file.

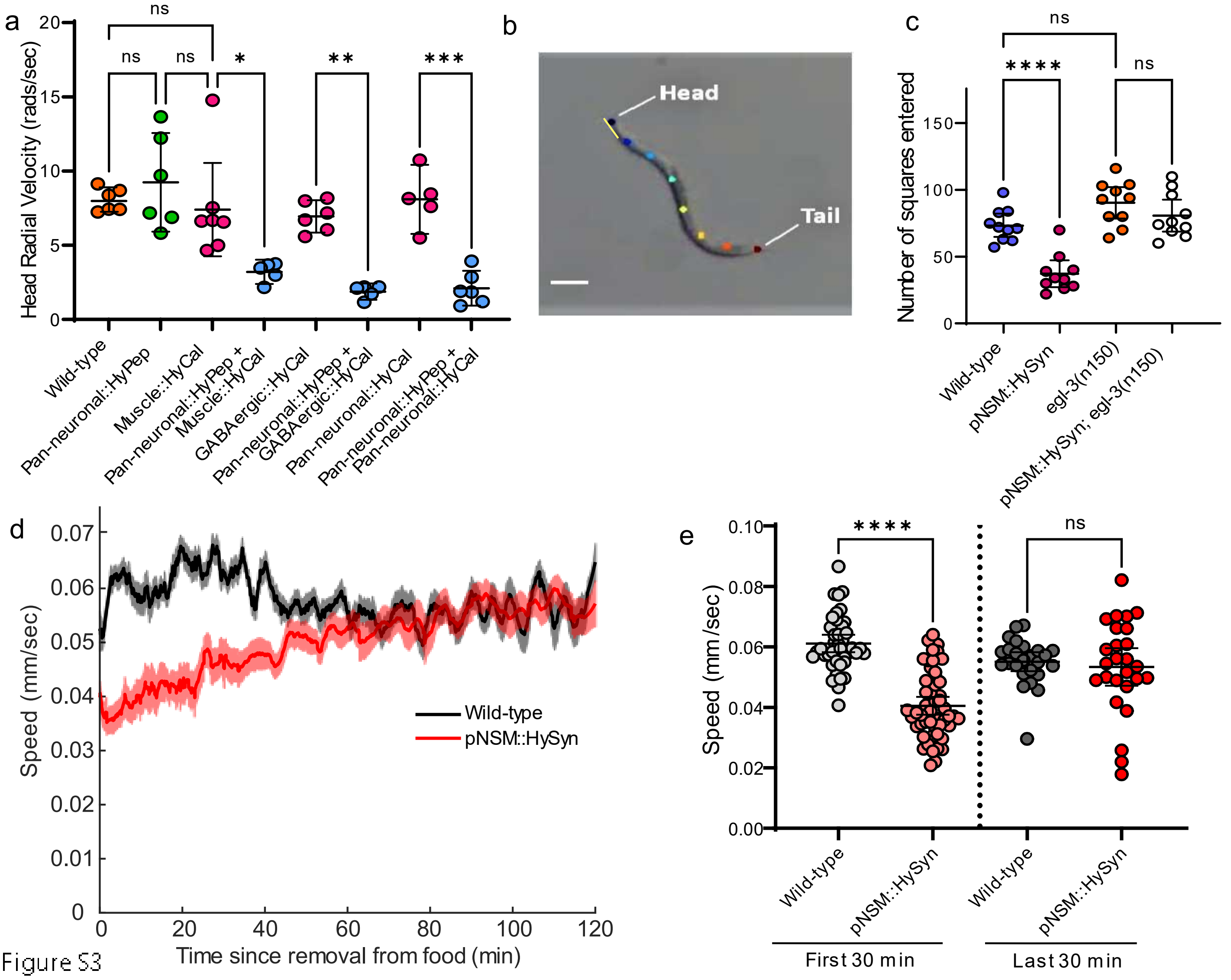

a
Head Radial Velocity (rads/sec)
ns
ns
ns
*
**
***
Wild-type
Pan-neuronal::HyPep
Muscle::HyCal
Pan-neuronal::HyPep + Muscle::HyCal
GABAergic::HyCal
Pan-neuronal::HyPep + GABAergic::HyCal
Pan-neuronal::HyCal
Pan-neuronal::HyPep + Pan-neuronal::HyCal
b
Head
Tail
c
Number of squares entered
ns
****
ns
Wild-type
pNSM::HySyn
egl-3(n150)
pNSM::HySyn; egl-3(n150)
d
Speed (mm/sec)
Time since removal from food (min)
Wild-type
pNSM::HySyn
e
Speed (mm/sec)
****
ns
Wild-type
pNSM::HySyn
Wild-type
pNSM::HySyn
First 30 min
Last 30 min

Figure S3

Supplementary Figure 3. Reconstitution of HySyn modulates animal behavior. a, Quantification of *C. elegans* movement by using swimming assays. Indicated tissue-specific promoters were used to drive the expression of the pre- and postsynaptic components of HySyn (HyPep and HyCal), as indicated, and worm movement was calculated by quantifying the head-radial velocity as measured in radians/second ('rads/sec') (p=0.0188 for pan-neuronal::HyPep; muscle::HyCal (n=6) vs. muscle::HyCal (n=6), p=0.0035 for pan-neuronal::HyPep; GABAergic::HyCal (n=5) vs. GABAergic::HyCal (n=6), p=0.0004 for pan-neuronal::HyPep; pan-neuronal::HyCal vs. pan-neuronal::HyCal (n=6)). **b,** Image of a L1-staged animal swimming, with superimposed points which enable measurements of body position during the swimming assays (SV2 and SV3). DeepLabCut was used to train a neural network to identify worm postures and superimpose the indicated points[28, 29]. See also Supplementary Video S3 highlighting body position identification and head vector quantification. **c**, Data from behavioral migration assays for indicated genotypes. These assays were conducted in parallel to those in Fig 4. 'pNSM::HySyn' refers to reconstitution of HyPep in the NSM neuron and HyCal in body wall muscles. Note that these animals (red) have decreased locomotion (more dwelling behavior) as compared to the wild-type (blue), suggesting an additive neuromodulatory effect of HySyn reconstitution, above that of serotonin, when the animals encounter food, likely due to the co-release of serotonin and HyPep from NSM during food encounters (p<0.0001 for pNSM::HySyn vs wild-type). In the background of a mutant gene necessary for neuropeptide processing, *egl-3(n150)*, the locomotion suppressing effects of pNSM::HySyn are abolished (white) (n=10 independent biological replicates per genotype measured over one experiment). **d,** Quantification of the mean speed of animals over the course of a 120-min long migration assay conducted in the absence of food, and immediately after the animals were removed from food. pNSM::HySyn animals exhibit an extinction of the food-induced HySyn phenotype, with a linear increase in mean speed over time before plateauing at the level of wild-type mean speed at 70 min. Bold lines (red, black) represent the mean speed and the shaded regions (red, black) represent a 95% confidence interval. **e,** Quantification of the mean speed of animals during the first (light gray and red) and last (dark gray and red) 30 min of a 120-min migration assay conducted in the absence of food (p<0.0001 for 'First 30min' pNSM::HySyn (n=57) vs wild-type (n=44)). In panels **a, c,** and **e,** an ordinary one-way ANOVA followed by a Tukey's multiple-comparison *post hoc* test was used to compare the means of each group. * indicates p<0.05, "ns" indicates that no statistically significant difference was observed. Error bars (black) represent the mean of each group and a 95% confidence interval. In all graphs, each dot in the graph represents an individual worm (an independent biological replicate). Source data are provided as a Source Data file.
