# Supplementary material for: A genetically encoded tool for reconstituting synthetic modulatory neurotransmission and reconnect neural circuits in vivo
Source: Nat Commun. 2021 Aug 9;12:4795. doi: 10.1038/s41467-021-24690-9 (PMC8352926; doi:10.1038/s41467-021-24690-9)
Supplement: Supplementary file 3 — Description of Additional Supplementary Files [file 41467_2021_24690_MOESM3_ESM.pdf]

Description of Additional Supplementary Files

File Name: **Supplementary Data 1**
Description: The name of constructs, the vectors (for building the constructs), and sequences used in this study.

File Name: **Supplementary Data 2**
Description: List of strains and the corresponding genotypes used in this study.

File Name: **Supplementary Data 3**
Description: List of primers used for genotyping mutant *C. elegans* strains used in this study.

File Name: **Supplementary Movie 1**
Description: Swimming behavior in transgenic *C. elegans* expressing the synthetic neuron-to-muscle HySyn connection. Order of display: Wild-type *C. elegans*, Neuromuscular HySyn, *egl-3(n150)* animals, Neuromuscular HySyn in *egl-3(n-150)* background. Scale bar is 200μm. 'Neuromuscular HySyn' is the same HySyn configuration as 'rab-3::HyPep; myo-3::HyCal' noted in SV2.

File Name: **Supplementary Movie 2**
Description: Swimming behavior in transgenic *C. elegans* expressing the respective pre- and postsynaptic HySyn components as ordered left to right in Fig S3a. Real-time read-outs of the quantification of head-radial velocity measured in radians (top left). Order of display in video: 1) Wild-type (N2), 2) pan-neuronal::HyPep (rab-3::HyPep), 3) muscle::HyCal (myo-3::HyCal), 4) pan-neuronal::HyPep + muscle::HyCal (rab-3::HyPep; myo-3::HyCal), 5) GABAergic::HyCal (unc-47::HyCal); 6) pan-neuronal::HyPep + GABAergic::HyCal (rab-3::HyPep; unc-47::HyCal, 7) pan-neuronal::HyPep + pan-neuronal::HyCal (rab-3::HyPep; rab-3::HyCal). 'Pan-neuronal::HyPep + muscle::HyCal' (rab-3::HyPep; myo-3::HyCal) is the same HySyn configuration as 'Neuromuscular HySyn' noted in SV1.

File Name: **Supplementary Movie 3**
Description: 10 x slower video clip (3 fps) of SV2 of swimming behaviour in wild-type *C. elegans* to demonstrate the technique used for measuring head-radial velocity over the course of swimming assays.
