# Supplementary material for: A genetically encoded tool for reconstituting synthetic modulatory neurotransmission and reconnect neural circuits in vivo
Source: Nat Commun. 2021 Aug 9;12:4795. doi: 10.1038/s41467-021-24690-9 (PMC8352926; doi:10.1038/s41467-021-24690-9)
Supplement: Supplementary file 10 — Reporting Summary [file 41467_2021_24690_MOESM10_ESM.pdf]

nature research

Corresponding author(s): Josh D. Hawk and Daniel A. Colón-Ramos

Last updated by author(s): May 25, 2021

Reporting Summary

Nature Research wishes to improve the reproducibility of the work that we publish. This form provides structure for consistency and transparency in reporting. For further information on Nature Research policies, see our Editorial Policies and the Editorial Policy Checklist.

Statistics

For all statistical analyses, confirm that the following items are present in the figure legend, table legend, main text, or Methods section.

n/a	Confirmed	
☐	☒	The exact sample size (*n*) for each experimental group/condition, given as a discrete number and unit of measurement
☐	☒	A statement on whether measurements were taken from distinct samples or whether the same sample was measured repeatedly
☐	☒	The statistical test(s) used AND whether they are one- or two-sided<br>*Only common tests should be described solely by name; describe more complex techniques in the Methods section.*
☒	☐	A description of all covariates tested
☐	☒	A description of any assumptions or corrections, such as tests of normality and adjustment for multiple comparisons
☐	☒	A full description of the statistical parameters including central tendency (e.g. means) or other basic estimates (e.g. regression coefficient) AND variation (e.g. standard deviation) or associated estimates of uncertainty (e.g. confidence intervals)
☐	☒	For null hypothesis testing, the test statistic (e.g. *F*, *t*, *r*) with confidence intervals, effect sizes, degrees of freedom and *P* value noted<br>*Give P values as exact values whenever suitable.*
☒	☐	For Bayesian analysis, information on the choice of priors and Markov chain Monte Carlo settings
☒	☐	For hierarchical and complex designs, identification of the appropriate level for tests and full reporting of outcomes
☒	☐	Estimates of effect sizes (e.g. Cohen's *d*, Pearson's *r*), indicating how they were calculated

Our web collection on statistics for biologists contains articles on many of the points above.

Software and code

Policy information about availability of computer code

Data collection	SutterPatch v2.0.3 (build 216), uManager vl.4, DeepLabCut (version 2.2b8), using IC Capture Easy Image Acquisition software (Version 2.5.1525.3931, 64 bit). Full code: https://github.com/colonramoslab/Hawk-et-al.-HySyn-2021.git or DOI: 10.5281/zenodo.4782623
Data analysis	SutterPatch v2.0.3 (build 216), Matlab R2 019b, LabView 2014, MagatAnalyzer (v1.0, https://github.com/samuellab/MAGATAnalyzer), DeepLabCut (version 2.2b8), ImageJ/FIJI (v1.52), Adobe Illustrator (2020 24.3.0), Full code: https://github.com/colonramoslab/Hawk-et-al.-HySyn-2021.git or DOI: 10.5281/zenodo.4782623

For manuscripts utilizing custom algorithms or software that are central to the research but not yet described in published literature, software must be made available to editors and reviewers. We strongly encourage code deposition in a community repository (e.g. GitHub). See the Nature Research guidelines for submitting code & software for further information.

Data

Policy information about availability of data

All manuscripts must include a data availability statement. This statement should provide the following information, where applicable:

- Accession codes, unique identifiers, or web links for publicly available datasets
- A list of figures that have associated raw data
- A description of any restrictions on data availability

Data Availability Statement. The source data generated in this study (plasmids and vectors) are available at Addgene (Deposit #78628). All relevant sequences are available in Supplementary table 1. The data for the head thrashing assays (Fig S3a) is available on GitHub (DOI: 10.5281/zenodo.4782623). All other data that support the findings of this study are reported within the manuscript.

Field-specific reporting

Please select the one below that is the best fit for your research. If you are not sure, read the appropriate sections before making your selection.

☒ Life sciences ☐ Behavioural & social sciences ☐ Ecological, evolutionary & environmental sciences

For a reference copy of the document with all sections, see nature.com/documents/nr-reporting-summary-flat.pdf

Life sciences study design

All studies must disclose on these points even when the disclosure is negative.

Sample size	Because the parameters of this novel synthetic synapse were unknown at time of study design, we could not assess the number of replicates necessary for most analyses. A power analysis was conducted before the on-food migration assay took place, and an n = 10 was decided upon based on that, and, to allow for all animals of all genotypes tested to be fully analyzed within experimental time frames.
Data exclusions	Electrophysiological data was only obtained if a stable patch was achieved, and no data were excluded from Fig 3, 4, or S3. The experiment in Figure 2b-d was designed to test the reproducibility, at a population level, of the HySyn response, which we observed in 34% of the cells examined. Similarly, in the data acquired for Figure 2f,g we observed responses in only 45% (14/31) of the cells, as discussed in the text. Because this experiment was designed to assess within-subject reproducibility of these responses, we excluded those samples lacking a response (with <3 SD above baseline) from follow-up analysis in Figure 2f,g, although they are reported in the statistic above within the text.
Replication	Replication of electrophysiology experiments is shown in Supplementary Figure S2. The individual means of responses for independent biological replicates are shown in Figure S2b-d, where each dot represents the mean of technical replicates (e-g) for each biological replicate. As shown, the electrophysiological effect, like the calcium imaging effect, was replicated in approximately half of the examined cells. Similarly, in calcium imaging (Figure 2b-d) a robust calcium response was observed in 34% of cells, or in 45% of cells in another set of biological replicates.<br><br>For the C. elegans migration assays conducted in Figure 3k-l, S3d-e, and 4a-d, independent animals of each genotype were tested on identically prepared substrates, where each animal constitutes an independent biological replicate. All animals were chosen at random from the respective mutant population of animals and/or based solely on the expression of fluorescent markers and not based on any observable phenotypes (i.e paralysis). Similarly, for the head thrashing experiments conducted in Figure S3a, at least 5 animals from the respective groups were tested. Each animal within each group constitutes an individual biological replicate taken otherwise randomly from the population.
Randomization	For the cell culture-based experiments, the initial cell populations were split into groups by a random nature of parallel splitting and treatment with distinct plasmid transfection. We then randomly sampled cells from each population in electrophysiology and calcium imaging based solely on reporter transgene expression (GFP or mCherry). Groups were randomly sampled over time and these experiments are within-subject, minimizing the influence of covariates.<br><br>For the experiments involving C. elegans (Figures 3k-l, S3d-e, and 4a-d), the animals that were assayed were chosen at random from a population of animals based solely on fluorescent reporter expression. Animals were assayed on identically prepared solutions or substrates made in a single batch and analysis was performed in parallel in a single session including all groups, thus minimizing the influence of covariates.
Blinding	Use of fluorescent markers for targeting manipulations generally precluded wholly blind data acquisition. Yet, analysis was performed blind to group or, in the case of paralysis migration assays (Fig 3k-l) or off-food migration assays (S3d,3), through a fully automated pipeline. For the on-food migration assays (Fig 4b,d, S3c), all animals were picked at random from respective populations based solely on fluorescent reporters, and, at the time of scoring and data entry, all group identifying information was blinded to the experimenter.

Reporting for specific materials, systems and methods

We require information from authors about some types of materials, experimental systems and methods used in many studies. Here, indicate whether each material, system or method listed is relevant to your study. If you are not sure if a list item applies to your research, read the appropriate section before selecting a response.

Materials & experimental systems

n/a	Involved in the study	
☒	☐	Antibodies
☐	☒	Eukaryotic cell lines
☒	☐	Palaeontology and archaeology
☐	☒	Animals and other organisms
☒	☐	Human research participants
☒	☐	Clinical data
☒	☐	Dual use research of concern

Methods

n/a	Involved in the study	
☒	☐	ChIP-seq
☒	☐	Flow cytometry
☒	☐	MRI-based neuroimaging

Eukaryotic cell lines

Policy information about cell lines

Cell line source(s)	Neuro2A gifted from Zhao-Wen Wang (UConn). Original source citation: Neuro-2a (ATCC CCL-131).
Authentication	None
Mycoplasma contamination	Not tested
Commonly misidentified lines (See ICLAC register)	No commonly misidentified cell lines were used in the study.

Animals and other organisms

Policy information about studies involving animals; ARRIVE guidelines recommended for reporting animal research

Laboratory animals	C. elegans bristol N2. Males were used for necessary crosses. Hermaphrodites were used for all experimentation, and the age of animals is noted in the methods and figure legends too. In summary, all animals tested were in the "Young Adult" stage (synced as L4's in 18-24hrs in advance of assays, except those animals tested in the Head Thrashing Assay's, in which case L1 staged animals were used.
Wild animals	No wild animals were used in this study.
Field-collected samples	No field-collected samples were used in this study.
Ethics oversight	No ethical oversight is required for C.elegans research.

Note that full information on the approval of the study protocol must also be provided in the manuscript.
